# Signal design and courtship presentation coincide for highly biased delivery of an iridescent butterfly mating signal

**DOI:** 10.1111/evo.12551

**Published:** 2014-12-03

**Authors:** Thomas E White, Jochen Zeil, Darrell J Kemp, R Rodriguez, T Lenormand

**Affiliations:** 1 Department of Biological Sciences, Macquarie UniversityNorth Ryde, NSW, 2113, Australia; 3 Research School of Biology, ARC Centre of Excellence in Vision Science, The Australian National UniversityCanberra, ACT, 0200, Australia

**Keywords:** Color signals, Lepidoptera, private communication, sexual selection, ultraviolet

## Abstract

Sensory drive theory contends that signaling systems should evolve to optimize transmission between senders and intended receivers, while minimizing visibility to eavesdroppers where possible. In visual communication systems, the high directionality afforded by iridescent coloration presents underappreciated avenues for mediating this trade-off. This hypothesis predicts functional links between signal design and presentation such that visual conspicuousness is maximized only under ecologically relevant settings and/or to select audiences. We addressed this prediction using *Hypolimnas bolina*, a butterfly in which males possess ultraviolet markings on their dorsal wing surfaces with a narrow angular reflectance function. Males bearing brighter dorsal markings are increasingly attractive to females, but also likely more conspicuous to predators. Our data indicate that, during courtship (and given the ritualized wingbeat dynamics at these times), males position themselves relative to females in such a way as to simultaneously maximize three components of known or putative signal conspicuousness: brightness, area, and iridescent flash. This suggests that male signal design and display have coevolved for the delivery of an optimally conspicuous signal to courted females. More broadly, these findings imply a potential signaling role for iridescence itself, and pose a novel example for how signal design may coevolve with the behavioral context of display.

Examination of color-based traits, particularly the exaggerated signals thought to evolve under sexual selection (Andersson 1994), has informed our knowledge of fundamental ecological and evolutionary processes (e.g., Endler 1983; Maia et al. 2013). Such work has, in turn, been greatly facilitated by the consistent logical framework offered by sensory drive theory (Endler 1992). This theory emphasizes how broader contexts of signal generation, propagation, and reception can influence (i.e., drive) signaling systems along predictable phenotypic trajectories. In the case of visual signals, the relevant sensory context encompasses such features as ambient illumination and transmission environments, viewing backgrounds, and the visual/perceptual systems of ecologically relevant viewers (Endler 1992; Endler and Basolo 1998). Interpretations of sensory drive vary across the literature, and a common suggestion is that the framework be applied only to those contexts that directly deal with both signaling environments and receiver physiology (Stevens 2013). However, the original formulation of sensory drive theory (Endler 1992; Endler and Basolo 1998) implicates a wider breadth of signaling system features, encompassing factors such as the specifics of how signals are designed and presented. Sensory drive is also envisaged as a nonexclusive process, that is, to operate in conjunction with other processes (e.g., runaway sexual selection; Kirkpatrick 1982) to determine the trajectories of evolution in signaling systems (Endler 1992).

The phenotypic expression of most sexual signals is generally thought to represent a balance between the conflicting influences of sexual and natural selection (Endler 1983; Andersson 1994). These signals require high conspicuousness to effectively compete for mates (e.g., through the advertisement of mate or rival identity and/or quality), yet at the same time they need to maintain relatively low conspicuousness to predators. Although classical models of trait evolution under sexual selection (i.e., Fisherian, handicap/good genes, and direct benefits models) consider signal content (Johnstone 1995; Kirkpatrick 1996), sensory drive emphasizes the importance of signal design, transmission, reception, and perception (Endler 1992; Endler and Basolo 1998). In the case of visual signals, a general insight is that design features such as color, patterning, signal directionality, and polarization should coevolve with display behavior in ways that lead to increasingly specialized transmission (Endler 1992, 1993a). Existing tests of this idea have proceeded largely in systems involving pigment-based signals (Kemp et al. 2012), and dealt with issues such as when, where, and how such signals are displayed (e.g., Land 1993; How et al. 2007). One avenue that remains relatively understudied, however, both in the context of sensory drive and in studies of visual signaling more generally, is the potential for the directionality of structural color to bias visual signal delivery.

Structural colors arise via an interaction between incident light and the micro- or nanoscale architecture of a surface (Land 1972). Such colors feature extensively in sexual displays (e.g., the Peacock's train; Dakin and Montgomerie 2009), and contribute to some of nature's most striking visual signals (e.g., Vukusic et al. 2002; Schultz and Fincke 2009; Seago et al. 2009). Compared to pigment-generated colors, structural colors present a number of features that have interesting potential implications for their role and evolution as signals. First, such colors have the potential for extreme brightness and chromaticity, meaning that they can reflect a high amount of light overall or in select regions of the light spectrum, contributing to “rich” or “vivid” color. These characteristics are likely to furnish high signal conspicuousness (i.e., signal-to-noise ratios) under most viewing conditions. Second, structural color may facilitate the exploration of otherwise inaccessible areas of color space; that is, hues or degrees of chromaticity that are otherwise difficult to achieve by pigments (Vertesy et al. 2006). Third—and of direct interest to this study—is the fact that many (although not all) such signals are iridescent. This property refers to a change in apparent hue and/or brightness depending upon the angle at which the signaling surface is viewed (and/or illuminated). Such signals may only be visible over a relatively narrow range of viewing angles, in which case they are said to possess a narrow reflectance function (Vukusic et al. 2002; Stavenga et al. 2010). In these cases, precise geometries of light source, signaler, and receiver are necessary for signal transmission.

The narrow reflectance function of many structural colors provides novel opportunities for biasing signal transmission (Endler 1992). One such possibility is the use of precise behavioral displays that serve to direct the signal at its most conspicuous expression toward intended receivers. This hypothesis is based on coevolution between features of iridescent signal design, such as angular visibility or color flicker, and the components of behavior, such as display rate, body orientation, and relative signaler–receiver positioning, that determine when, where, and how the signal is transmitted. The potential for behavior to modify the appearance of iridescent signals has long been recognized (e.g., Poulton 1890; Endler 1983), but there have been surprisingly few attempts to quantify such effects. The most rigorous existing studies focus upon avian systems (Hamilton 1965; Dakin and Montgomerie 2009; Sicsu et al. 2013). At the same time, knowledge of iridescent signal design has greatly increased, particularly in insect systems (Seago et al. 2009; Kemp and Rutowski 2011), thereby offering a broader and potentially more tractable spread of taxa for examining this hypothesis.

Butterflies exhibit a diversity of color-producing mechanisms, high laboratory tractability, and often complex display behaviors (Stride 1956, 1957), making them ideal for exploring the evolution of iridescent signaling systems. Species with angularly restricted sexual signals, such as *Hypolimnas bolina* (the common eggfly), present special empirical opportunities. Males of this species express large spots of iridescent UV/violet color on their dorsal fore- and hind wings (Kemp and Macedonia 2006). These markings have a narrow angular reflectance function (i.e., they are “limited-view,” sensu Vukusic et al. 2002), being visible only from an approximately 20° range of above-wing viewing angles (Kemp and Macedonia 2006). Female *H. bolina* have been demonstrated to prefer males bearing brighter UV wing patches, under both flight cage and field conditions (Kemp 2007), which implies that males should endeavor to present their brightest signal during courtship. Males do indeed exhibit ritualized courtship behaviors (see below), but it is not presently known whether their behavior and positioning serves to transmit maximally bright UV (or some other signal feature that might affect signal perception, such as visible signal area, or color flicker; Rutowski et al. 2007).

In this study, we investigate whether the behavior of courting male *H. bolina* has evolved to maximize the conspicuousness of their iridescent signal (i.e., to transmit maximally bright UV; Kemp 2007), as seen from the perspective of a courted female. We approached this by first summarizing the complexity in both signal design and courtship behavior in terms of manageable components (including male wingbeat amplitude, vertical distance, and position on the horizontal plane relative to the female; see Materials and Methods). We then sought to empirically estimate all key parameters except for one (male position on the horizontal plane), which offered the basis for testing predictions. Our approach is summarized by the following three stages:

1. We first used a combination of regular and high-speed video to quantify three key components of male flight dynamics during courtship: wingbeat frequency, wingbeat amplitude, and vertical positioning relative to females. Here, we sought to characterize what male *H. bolina* do “on average” during courtship. We also measured the first two components for males in regular (noncourtship) flight.
2. We then used reflectance spectrometry to quantify how the male signal would appear to a female given a range of potential positions across a 1 m^2^ plane (arena) situated 200 mm below her (the average vertical positioning of a courting male, as obtained in stage 1; see Results). We quantified signal appearance at a grid of points across this arena assuming that a male situated at each point was flapping his wings according to the properties identified in step 1.
3. We then used the information gained through step 2 to generate predictions as to where males should position themselves during courtship to maximize signal transmission. These were subsequently compared to the observed positioning of a sample of courting males, as characterized using high-speed video, to test whether males do indeed achieve optimal signal transmission.

## Materials and Methods

### SEXUAL SIGNALLING IN *H. BOLINA*

Male *H. bolina* attempt to locate receptive females by establishing themselves at local vantage-points in the environment and investigating anything that flies nearby. If an actively mate-searching male locates a female, he will approach and pursue from a position beneath her, all the while performing a ritualized “fluttering” courtship display. Females adopt their own semiritualized flight during these times, which has the appearance of a high wingbeat frequency “hovering” flight, and maintain their height of between 1 and 3 m from the substrate (Stride 1956, 1957). The duration of these aerial displays ranges from a few seconds to several minutes, after which the female will either break off the engagement by maneuvering away (typically by ascending rapidly to heights in excess of 15 m; Edmunds 1969), or settle to allow mating.

### SPECIMEN PROVENANCE AND LABORATORY REARING

We conducted experiments on 32 individuals purchased as larvae from a commercial breeder located in Cairns, Queensland, and their laboratory-reared F1 (*N* = 52) and F2 (*N* = 38) descendants. The founding individuals were themselves the direct offspring of crosses between multiple field-collected individuals, which means it is highly unlikely that their behavior has been shaped by adaptation to mass rearing conditions. All butterflies were reared using standard husbandry protocols (Kemp 2007), using greenhouse-cultivated food plant (*Asystasia gangetica*), and under 26.0 ± 1.0^o^C and 14:10 L:D photoperiod. Pupae were allowed to develop at 29.0 ± 1.0^o^C. Emerged adults were transferred to an outdoor greenhouse (7.0 × 7.0 × 3.5 m height), which was loosely controlled to a temperature range of 21–29°C. Adults were provided *ad libitum* access to potted *Pentas lanceolata* and cotton wool saturated with a 1:10 solution of honey water. Twenty F1 males were killed immediately upon adult emergence for reflectance spectrometry, and their dimensions (wingspan, abdomen length and width, forewing size) recorded to the nearest 0.1 mm using digital callipers.

### QUANTIFYING MALE FLIGHT DYNAMICS

We used high-speed video to characterize male flight dynamics (wingbeat frequency and wing-sweep amplitude) during courtship and regular flight, as well as to estimate male and female x-y positioning on the horizontal plane during courtship. All video recording took place in the greenhouse under the conditions as previously described. Video was captured from May to July 2012 using two Casio (Casio Computer Company, Ltd., Tokyo, Japan) Exilim Ex-F1s recording at 300 fps and 512 × 384 resolution. Recording took place between 1000 and 1500 h under full sunlight, as males are extremely reluctant to court under cloudy conditions (Kemp 2007). The cameras were fixed to the greenhouse ceiling (∼3.5 m height) at a working distance of approximately 1–2 m to the courting butterflies. The cameras were periodically focused on a checkerboard at a distance of 1.5 m. Coordinate data were extracted from the high-speed footage using *digilite*, a program created in MATLAB (The MathWorks, Inc., Natick, MA) by Jan Hemmi and Robert Parker of the Australian National University. Through each frame of footage, we tracked the positions of each individual's head, the tip of each forewing, and the distal tip of the abdomen. We then calculated wingbeat frequency, wingbeat amplitude, and male–female x-y positioning on the horizontal plane using custom written MATLAB scripts (see Supplementary Methods in the Supporting Information). A total of 28 independent courtship sequences and 30 independent regular flight sequences were analyzed.

### QUANTIFYING VERTICAL MALE–FEMALE COURTSHIP DISTANCE

We videotaped 30 courtship sequences between April and June 2012 to estimate the average vertical distance between males and females during courtship. For this, we used a Panasonic (Panasonic Corp., Osaka, Japan) Lumix DMZ-FZ35 recording 25 fps at a resolution of 1280 × 720 pixels. The camera was set at a height of approximately 2 m in the corner of the greenhouse and all courtships were recorded from a lateral perspective, with an average distance of approximately 4 m between the camera and courting butterflies. We converted the courtship footage into image sequences, randomly selected 10 frames from each, and measured the vertical distance between the interacting female and male using ImageJ (Abramoff et al. 2004). Male forewing and abdomen dimensions were used to scale each image individually. A one-way analysis of variance (ANOVA) showed no effect of courtship sequence on vertical male–female distance (*F*_1, 29_ = 1.49, *P* = 0.056), so measures were pooled across courtships. Individuals were not tracked between courtships, which raises the possibility of pseudo-replication, in that some males may have contributed to more than one courtship sequence. However, recording involved approximately 50 males across three generations, which implies a low likelihood that any single individual contributed to multiple sequences.

### QUANTIFYING MALE SIGNAL DYNAMICS

We used an Ocean Optics (Ocean Optics, Inc., Dunedin, FL) USB-4000 spectrometer to capture reflectance spectra from a ∼5 mm diameter region at the UV-only edge of the spot on each dorsal wing surface of 20 males (see Fig. S1). The spectrometer was set to an integration time of 100 ms and to average 10 successive scans. Illumination was provided normal to the wing surface using a PX-2 pulsed xenon light source, which has high output over the range of 300–700 nm. Wing reflectance was measured relative to a magnesium oxide standard, as per prior work in this system (e.g., Kemp and Macedonia 2006).

**Figure 1:**
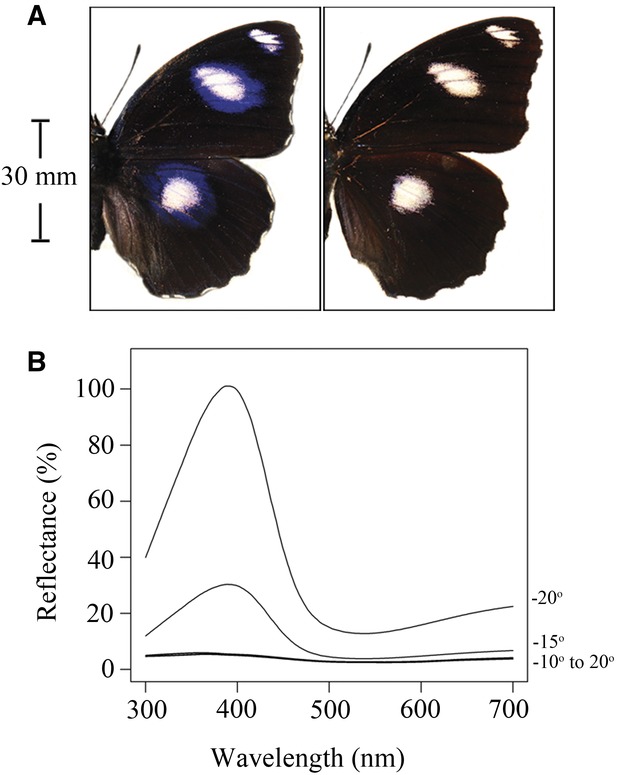
(A) Dorsal wing coloration of male *Hypolimnas bolina* as viewed from orientations conducive to seeing (left) or not seeing (right) the iridescent UV signal. (B) Representative angle-resolved reflectance spectra of the iridescent UV signal. Spectra were captured with the light source and probe normal to the wing surface (i.e., as a female would view it; see Results), and wings were rotated through angles –20^o^–20^o^ in 5^o^ increments, as indicated on the right *y*-axis. Spectra were averaged across fore- and hind wings at each orientation (*n* = 5).

To characterize how male position relative to female position affects signal appearance, we measured male wing reflectance as it would appear (to the female) from nine different viewing positions. We achieved this by manipulating the angle of the spectrometer's collector and rotating individual wings on a universal stage (see Figs. S1, S2). In each of the nine positions, we measured reflectance for each wing at five angular orientations along its proximal–distal axis: –40^o^, –20^o^, 0^o^, 20^o^, 40^o^. We also recorded the angle at which the iridescent signal turned “on” (defined as >10% peak reflectance amplitude in the 300- to 450-nm range; Fig. 1) for each wing at every position. For viewing orientations outside this range, wherein no UV reflectance is evident from the wing, we refer to the UV signal as being “off.” Due to the number of measurements required, we restricted the simulated courtship arena to a 1.0 m^2^ horizontal plane situated 200 mm below the female (see Results for justification of the 200-mm vertical distance). We also assumed no male body pitch, roll, or yaw during courtship flight, and negligible wing torsion. These assumptions are well founded empirically (Steppan 1996; Rutowski et al. 2007), and further substantiated in our analysis of male flight dynamics.

Three metrics were used to summarize iridescent signal conspicuousness: total reflectance (hereafter referred to as signal brightness), flash-effect, and signal area. Following Kemp and Macedonia (2006) and Kemp (2007), brightness was estimated as the mean of reflectance amplitudes in the 300- to 450-nm waveband, which represents the spectral region of maximum reflectance. Flash-effect represents the relative time the signal spends “on” versus “off” (as defined above) during a wingbeat, and is estimated by the proportion of the wingbeat for which the signal is off. Signal area was approximated for each viewing orientation simply as the number of wings for which UV iridescence was “on” (thus ranging from zero to four wings).

### MODEL TESTING

We generated testable predictions as to where males should position themselves during courtships by constructing six models of signal conspicuousness based on combinations of brightness, flash-effect, and signal area. As there are a number of ways in which a signal may conceivably be attractive to females, our models represent an attempt to capture what we hypothesize to be the most important features of the signal, both individually and in various combinations. Although all combinations of variables were considered, the final models actually tested were those which were considered the most biologically plausible. For this reason, we included signal brightness—a known correlate of attractiveness in this species (Kemp 2007)—as a key feature of all candidate models.

We considered the following models, which essentially formalize candidate hypotheses for what may constitute an attractive visual signal in this species: (i) brightness, (ii) brightness and signal area, (iii) brightness and flash-effect, (iv) brightness and flash-effect and signal area. These models were then used to visualize how the signal conspicuousness would vary with male positioning during courtship (as viewed by a female) by generating a series of contour maps. Each map essentially represents a prediction, expressed across a horizontal *x*–*y* plane, for how males should best position themselves relative to a female (centered at the coordinates *x* = 0, *y* = 0), to maximize the specific signal attribute(s) in question. These maps may also be mirrored about both axes and projected on a hemisphere to visualize male signal expression (centered on the hemisphere floor) from the entire overhead perspective. We generate such maps for the most highly supported models of signal conspicuousness (see Results) because they indicate how the signal may be broadcast more widely, that is, its potential appearance to eavesdroppers at positions other than that of a courted female.

We tested which model ([i]–[iv], as above) best fit actual male behavior during courtship by plotting all recorded male head coordinates (all standardized such that they were relative to the courted female's head, that is, with the position of the female centered to *x* = 0, *y* = 0 in the horizontal plane) onto the contour map for each model, then extracting the “signal intensity” scores (*z* values in the *x*–*y*–*z* coordinate system, where *z* is a nonspatial dimension representing the signal intensity—the “height” of contours) for every male *x*–*y* coordinate. These intensity scores may be interpreted as a measure of how a particular male has “scored” in an instant of time, in terms of the parameter(s) considered by the model. For example, regions of higher signal intensity for model (i) indicate that a courted female would see a brighter signal when a male is in that particular region of the horizontal plane; higher values in model (ii) indicate the presentation of a brighter signal of greater area, etc. The cumulative sum of these scores was then used to summarize total signal intensity delivered to a female viewer during each courtship sequence, and these values were then summed across sequences. We normalized these data so that signal intensity scores (i.e., cumulative *z*-axis value) were directly comparable across models (see Supplementary Methods in the Supporting Information for further details). The final product were four directly comparable values that represented the fit of the models against the empirical data on actual male positioning during the videotaped courtship sequences.

### STATISTICAL ANALYSES

We used an ANOVA to statistically compare male wingbeat frequency between regular and courtship flight. We used a generalized linear mixed model (GLMM) to compare male wingbeat peak amplitude between regular and courtship flight, and included flight type (“courtship” or “regular”) and wingbeat stroke (which may take the value “up” or “down,” depending on which part of the wingbeat cycle a given amplitude measure was recorded from; nested within flight type) as categorical predictors, and courtship sequence as a random categorical factor. We used a GLMM to test for differences in the cumulative *z*-axis values (i.e., realized signal intensity) generated under each of the four putative models of signal conspicuousness (models [i]–[iv], as above); that is, given the observed *x*–*y* positioning of males during their courtship sequences (relative to a female centered at *x* = 0, *y* = 0), which model generated the highest value for cumulative signal intensity. This could also be thought of as a test for which model of signal conspicuousness best predicted male positioning during courtship. We included signal conspicuousness model as a fixed categorical predictor (coded 1–4 for models [i]–[iv]), cumulative signal intensity scores as the dependent variable, and individual courtship sequence as a random categorical factor. A Tukey's post-hoc honesty test was used to test for specific differences between signal conspicuousness models ([i]–[iv]). Parametric assumptions were confirmed for all datasets, with data transformed where necessary (and as stated in the Results). All analyses were conducted using Statistica version 10.0, and means are reported ± SE throughout.

## Results

### FLIGHT DYNAMICS

Male wingbeat dynamics consistently differed between courtship and regular flight. During courtship, males beat their wings at a higher frequency (11.1 ± 0.34 Hz; *F*_1, 55_ = 85.4, *P* < 0.001) and over a narrower angular range (i.e., with a smaller peak amplitude; 27.1 ± 0.41^o^; *F*_1, 791_ = 1664, *P* < 0.001) than during regular flight (frequency = 7.4 ± 0.23 Hz; peak amplitude = 56.6 ± 0.49^o^). Linear mixed modeling revealed no effect of courtship sequence (*F*_29, 791_ = 1.21, *P* = 0.211) or stroke type (*F*_2, 791_ = 2.11, *P* = 0.122) on peak wingbeat amplitude (square-root transformed). We also found that courting males positioned themselves, on average, at a vertical distance of 195 mm below females (95% CI = 187–201 mm), which we used to define 200 mm as an estimate for average male–female vertical courtship distance in subsequent analyses.

### COURTSHIP POSITIONING IN THE HORIZONTAL PLANE

**Figure 2:**
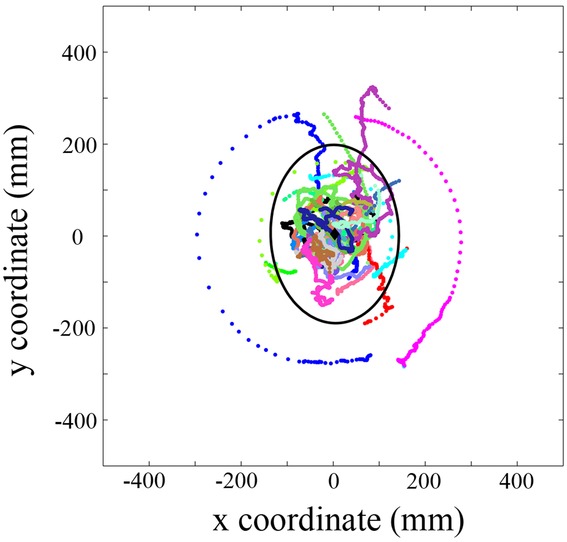
The positioning of male *H. bolina* relative to females (centered at *x* = 0, *y* = 0) as measured from 28 courtship sequences. Each sequence is shown in a unique color. The mean male coordinate on the horizontal plane is given (black diamond; mean ± SEM—*x* = 33.49 ± 7.58 mm, *y* = 44.0 ± 10.5 mm) along with its 95% confidence ellipse, which indicates a perimeter within which courting males spend 95% of their time. Male positions are represented at a temporal resolution of 3.33 msec.

During courtship, males spend the majority of their time almost directly underneath females in the horizontal plane. Plotting the male courtship position coordinates (all standardized as relative to a courted female head position at *x* = 0, *y* = 0 in this horizontal plane) revealed that males spend 95% of their time within an ellipse of approximately 200 × 400 mm (Fig. 2), centered around an average position of *x* = 33.4 ± 7.6 and *y* = 44.0 ± 10.5 mm (i.e., very slightly in front and to the right of the female's head).

### SIGNAL DYNAMICS

Based upon the analysis of male positioning and wingbeat amplitude during courtship, we restricted our subsequent assessment of male UV signal dynamics to a vertical male–female distance of 200 mm, a –20^o^–20^o^ range of wing angles, and a 1.0 m^2^ arena on the horizontal plane.

**Figure 3:**
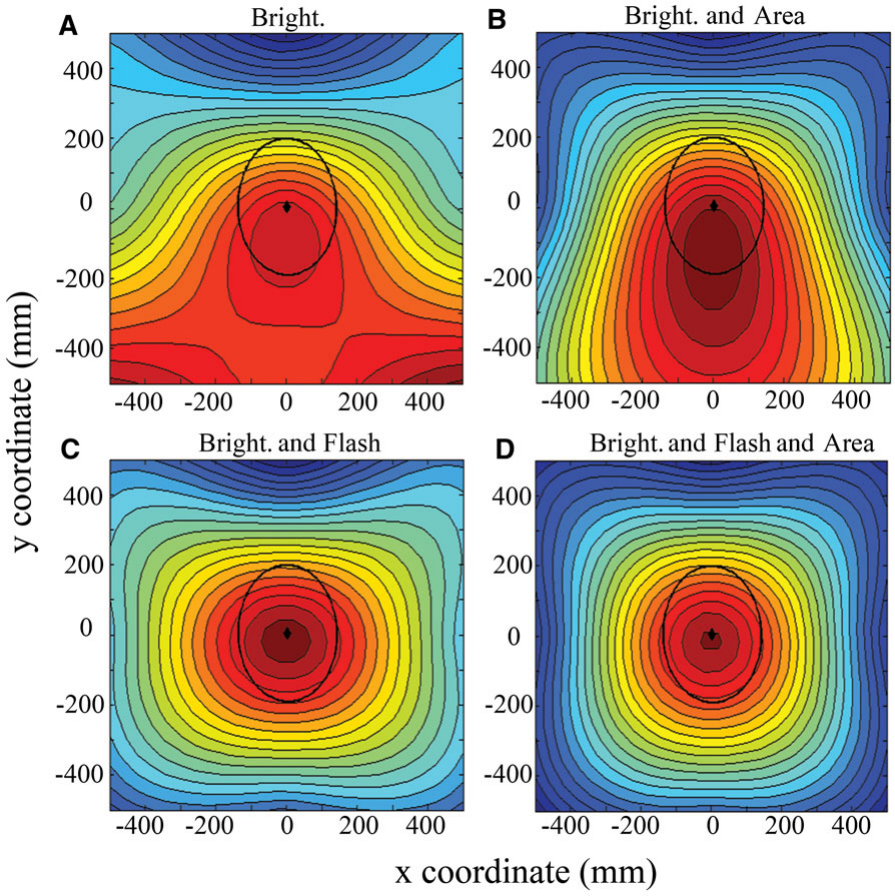
Summary of UV signal appearance relative to male positioning as viewed by a courted female (centered at *x* = 0, *y* = 0) and according to each candidate model of signal conspicuousness. The average positioning of males during courtship is indicated (black diamond) along with its 95% confidence ellipse. The color coding on each contour map represents a prediction for where males should position themselves if they are to maximize signal intensity (according to the signal attributes considered by each model). Normalized signal intensity is represented from low (blue) to high (red). Signal measurements made for the scenario where males are flying 200 mm below the female with a wingbeat amplitude of –20^o^–20^o^, as derived through assessment of actual courting males. “Bright,” UV brightness; “Area,” signaling area (defined by how many wing patches are visible); “Flash,” flash-effect, the proportion of a single wingbeat in which UV is not visible (i.e., the “brevity” of the UV flash per wingstroke).

The four models of signal conspicuousness produced qualitatively similar predictions, such that if males are attempting to maximize either (i) brightness, (ii) brightness and signal area, (iii) brightness and flash-effect, or (iv) brightness and flash-effect and signal area, then they should position themselves directly underneath or slightly behind the female during courtship. This is indicated by the high-intensity (i.e., “redder”) contour areas in Figure 3.

**Figure 4:**
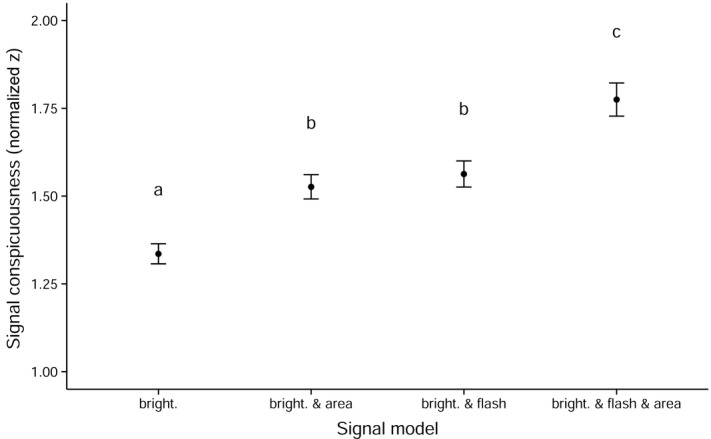
Summary of male performance in delivering a high-intensity signal according to each model of putative signal conspicuousness. Shown is the mean ± 95% confidence interval. Model descriptors along the *x*-axis are as described for Figure 3. Different letters above each box designate significant (α = 0.05) differences based on post-hoc comparisons using Tukey's HSD.

**Figure 5:**
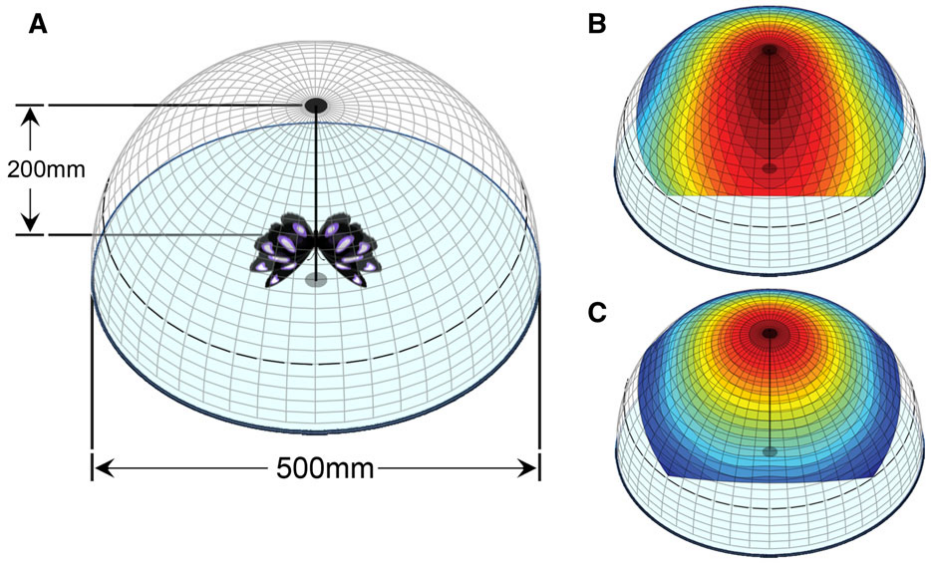
Hemispherical representations of male UV signal conspicuousness according to viewing orientation. (A) The hemispheres represent what a viewer would see of a courting male positioned as shown and flying with the average ritualized dynamics of courtship flight (i.e., a wingbeat frequency of ∼11 Hz and a wingsweep amplitude of ∼40°). The models in (B) and (C) were constructed as per Figure 3, then mirrored about both axes and projected onto the viewing hemisphere. Panel (B) indicates the model color coded for the signal features of UV brightness, area, and flicker, whereas panel (C) indicates a more conservative model of signal conspicuousness based only on UV brightness and area. Normalized signal intensity is represented from low (blue) to high (red).

**Figure 6:**
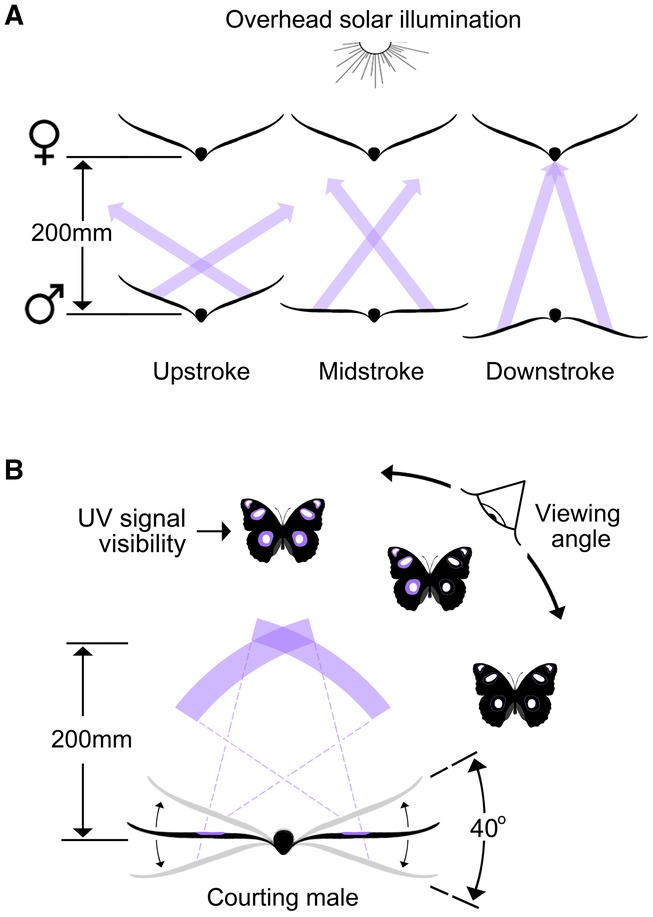
Summary dynamics of male UV visibility during courtship. Panel (A) traces the angular visibility of maximally bright UV reflectance (violet arrows) from the wings of a courting male over a complete wingstroke. A female situated 200 mm overhead would see this bright UV flash only at the point of the male's downstroke, but from all four wings simultaneously. Male UV would be invisible to the female for most of the remainder of each wingstroke, contributing a distinct series of flashes if this geometry can be maintained by the male. Panel (B) expresses the same courting male scenario but represents the visibility of maximally bright UV as a function of viewer orientation (and summarized for a viewing distance of 200 mm). The violet bands trace the orientations for UV visibility during a single male wingsweep, and the butterfly icons indicate which (of any) UV patches would be visible (indicated for viewers off to the right-hand side only). UV reflectance from both wings would be visible from overhead orientations (where the violet bands intersect), whereas viewers from more oblique angles would see UV from only one wing (one violet band visible) or from neither (no bands visible). Both panels assume identical “plane of depth” for the signaling male and viewer, and directly overhead illumination. Note that both panels summarize the visibility of maximal signal brightness—some UV would be visible at orientations immediately outside those indicated (i.e., ± ∼5° of the violet bands), however brightness declines extremely rapidly as viewing angle departs from the optimum (see Fig. 1).

We next analyzed “realized” signal conspicuousness as it would appear to females, based on the actual positioning of males during courtship and according to the four models explained above. The GLMM indicated a significant effect of both courtship sequence (*F*_27,135_ = 7.9, *P* < 0.001) and model (*F*_1,135_ = 476.9, *P* < 0.001) upon signal intensity score. The sequence effect indicates that male positioning during some courtship sequences was superior to others in terms of maximizing signal intensity. We therefore retained this factor in the model (as a random effect) to account for such variance. Given our primary interest in average male performance across models of signal conspicuousness, we focus hereafter on the main effect of “signal model.” Post-hoc multiple comparisons revealed highly significant differences in mean signal intensity scores across all models except for the contrast of “brightness and area” against “brightness and flash-effect” (Fig. 4). The single model of signal conspicuousness that best predicted male courtship behavior was that of “signal brightness and signal area and flash-effect” (Figs. 3D, 4, 5). Hence, males behave in such a way during courtship as to simultaneously maximize signal brightness, area, and flash-effect (by *minimizing* the period throughout a wingbeat cycle in which the signal is “on,” as defined above). In the courtship position (i.e., with males 200 mm directly below a courted female; Fig. 2), the male signal turns “on” at an angle of –12.05^o^ ± 0.63^o^. Thus, a female situated immediately above a courting male would experience a maximally bright signal from all four wings simultaneously (Fig. 6B), but only at the bottom of the male's downstroke (Fig. 6A). The male UV across all wings would then be invisible to the female for the remainder of each wingstroke (Fig. 6A), which would result in a distinct series of flashes for as long as this geometry can be maintained by the courting male.

## Discussion

Sensory drive has proven a valuable framework for interpreting features of visual signal design (Endler and Thery 1996; Kemp et al. 2009), but here we apply it explicitly to the design and presentation of an iridescent ornament—the highly directional UV wing coloration of male *H. bolina*. Our central finding is that, given the ritualized dynamics of male courtship flight in this species (Rutowski 1992), individuals position themselves beneath females in a way that simultaneously maximizes several components of putative signal conspicuousness—namely UV brightness, visible area, and flash-effect. Although essentially correlative, the close fit between the observed and a priori predicted male positioning on the horizontal plane (Fig. 3D) is consistent with a coevolutionary link between signal design and behavioral presentation (Endler 1992; Endler and Basolo 1998). Two features of our results are particularly noteworthy. First, individual males show a distinct behavioral shift between courtship and regular flight, characterized by a higher wingbeat frequency and shallower wingbeat amplitude during courtship. In this way, courting males essentially restrict the solid angle (i.e., the two-dimensional angle in three-dimensional space) over which their UV signal is broadcast relative to other flight situations, thereby enhancing signal transmission to females (Fig. 5). Second, courting males deliver a sharply flashing signal by positioning themselves relative to females in a way which minimizes the proportion of the wingbeat cycle during which maximally bright UV is visible to the female (Fig. 6). This implies a potential role for the iridescent flash effect itself in signal transmission, and potentially in contributing to the female's perception of male attractiveness. We discuss these points and other aspects of our results in relation to the broader literature on sexual signaling, iridescence, and sensory ecology.

Our findings that male courtship behavior enhances the likely perceived brightness of their UV signal (Fig. 3) is consistent with the known preference of female *H. bolina* for brighter UV males (Kemp 2007). Courting males also maximize signal area, in the sense that the UV patches on all four wings would often be simultaneously visible to females (Fig. 6B), which agrees with reports for other butterflies with similar mating signals (i.e., *Colias eurytheme*; Rutowski et al. 2007). Intriguingly, male courtship behavior also simultaneously maximizes the flash-effect, by minimizing the amount of time during each wingbeat cycle in which the signal is visible. Here, it is important to distinguish between the temporal *duration* of the UV flash event within a wingbeat (the property that we have measured and refer to as “flash-effect”), versus the *frequency* of the flash event, which is dependent only on wingbeat frequency. The ritualized flight of males during courtship is such that wingbeat frequency is regulated at ∼11 Hz; hence, a UV flash event would be delivered to females roughly 11 times per second. Our data show that (for this wingbeat frequency) the duration of successive UV flash events is minimized, each lasting approximately one-quarter of a wingbeat, or ∼23 msec, and separated by ∼66 msec. Still briefer UV flashes could be delivered through higher wingbeat frequencies, but it is difficult to assess the role of other potentially important constraints such as flight physiology and aerodynamics.

The perceptual relevance of flashing or strobe effects is well known (von Grünau et al. 1999; Schultz and Fincke 2009; discussed below). Such signals have long been thought to evoke super-normal stimulatory responses in butterflies (Magnus 1958; Vukusic et al. 2002), but there is only one explicit test of this hypothesis. By manipulating an artificial model, Magnus (1958) showed that male fritillary butterflies *Argynnis paphia* prefer stimuli that flash at speeds increasing up to the point where the eye's flicker-fusion rate is reached (∼100 Hz; Rutowski 2003). This result, coupled with our present findings that male *H. bolina* shift to shallower, higher frequency wingbeats during courtship, poses the working hypothesis that faster signal flashes may be an important constituent of signal attractiveness. As noted above, it will also be crucial to consider the aerodynamic constraints of flight (Srygley 2007), which are likely to determine the upper limits to how fast flashing stimuli can realistically be delivered.

Although the commonality of morphological adaptations for extreme iridescence suggest a signaling function for dynamic, directional colors (Vukusic et al. 2002; Stavenga et al. 2010), our knowledge of the adaptive significance of iridescence per se in sexual signaling is limited. Schultz and Fincke (2009) studied the directional, flashing wingbands of the giant damselfly *Megaloprepus caerulatus*, suggesting that they facilitate the long-range detection of conspecifics across forest light gaps. Long-range signaling of this nature is unlikely in *H. bolina* because males are the pro-active sex in mate location, and spend most of their time perching at mate location sites with wings closed (Rutowski 1992). Research in *H. bolina* has largely ruled-out a role for the iridescent male UV in male–male competition (Rutowski 1992; Kemp and Macedonia 2006), instead finding convincing evidence for a role in female mate preference (Kemp 2007). Similar findings in other butterflies with similar iridescent wing markings have prompted exploration into how these traits may signal mate quality. Interestingly, the brightness and/or narrow reflectance function of iridescent UV has been shown to depend upon individual condition in several coliadine species, including *C. eurytheme* (Kemp et al. 2006; Kemp and Rutowski 2007) and *Eurema hecabe* (Kemp 2008a). Such coloration is likely to act as a lifetime indicator of nutritious and thermally stable juvenile environments in these species (or of the genes for choosing appropriate juvenile environments; Kemp and Rutowski 2007). Notably, *H. bolina* and *C. eurytheme* share a similar type 1 ridge-lamellar architecture in which brightness and flash duration are mediated by potentially separate, although developmentally correlated, microstructural features (Kemp et al. 2006; White et al. 2012). In this sense, interindividual variation in flash duration may provide an additional axis of information to female butterflies regarding male phenotypic or genetic quality, as could the consistency of reflectance between left and right wings. Evidence is mounting across many taxa for the condition dependence of structurally colored sexual ornaments (e.g., Lim and Li 2007; Taylor et al. 2011), and for their role in determining mate attractiveness (Kemp 2008b; Lim et al. 2008; Kemp and Rutowski 2011).

Given the angular nature of the male UV signal, it is also important to consider that conspicuousness will ultimately be determined by not only the viewer's position, but also the position of the sun. Although courting males endeavor to control their positioning relative to females (Fig. 3), the haphazard flight orientations of courted females may make it difficult for males to simultaneously account for relative sun position. Nevertheless, the possibility exists for males to subtly adjust flight characteristics such as pitch, yaw, and roll under situations when the sun is lower in the sky, or even when they perceive their position as being lateral to the female. We cannot assess such finer scale effects here because courtship assessments were conducted from late morning to early afternoon, when the sun was largely overhead. However, this could be addressed using high-speed courtship footage captured outside of these times, or in an artificial setting with adjustable point-source illumination. In broader terms, it clearly stands that males should seek to bias signal transmission by courting selectively under direct sunlight, which would maximize the magnitude of the UV flash effect. Under overcast skies, the more diffuse illumination (Endler 1992, 1993b) would engender the visibility of much duller UV from a broader range of viewing angles, with a greatly reduced flash-effect. This implies that courting males may appear less attractive to females at such times—especially if signal flash is an important constituent of attractiveness. This is consistent with limited available evidence that males in the wild are reluctant to engage mates under cloudy skies (Rutowski 1992).

Sexual signaling systems are subject to the dual challenge of maximizing signal transmission to conspecifics (within prevailing constraints) while minimizing detection by visually orienting predators (Endler 1992; Zuk and Kolluru 1998). Theory predicts that such signals should, where possible, be designed and/or selectively broadcast to achieve a degree of privatization (Endler 1993a). This prediction has been solidly supported by demonstrations across a range of taxa for how sexual signals exploit differences in the visual physiology of predators and prey (e.g., Cronin et al. 2003; Cummings et al. 2003; Sweeney et al. 2003; Douglas et al. 2007; Siebeck et al. 2010). Poulton (1890) suggested over a century ago that privatization may also be effected by coupling a strongly directional signal with precise behavioral delivery, but this hypothesis has been rarely and/or indirectly addressed (e.g., Hamilton 1965; Stiles 1982; Dakin and Montgomerie 2009, 2013). Although not designed to bear squarely on this issue, our study indicates how maximally bright UV reflectance from multiple wing patches can only be seen from a very restricted viewing geometry (i.e., directly overhead a courting male *H. bolina*; Figs. 5B, 6). Unless positioned directly overhead or slightly anterior to a courting male (Fig. 5), a viewer is likely to see a relatively fleeting flash of less than maximally bright UV, and from a maximum of two wing patches at once (Fig. 6B). Outside of courtship, such as when males are dispersing, defending territories, or foraging for nectar, their higher wingbeat frequencies and broader wing sweep amplitudes would generally engender more fleeting UV flashes from a broader range of viewing orientations. Further, because eavesdroppers such as avian predators would likely view such males from haphazard and shifting orientations, they would rarely see the consistently flashing UV signal that males seek to deliver to females (Fig. 4C, D). These arguments are broadly consistent with Poulton's hypothesis, but a firm test of this idea would require dedicated enquiry into signal conspicuousness in relation to the likely viewing orientations of eavesdroppers.

Our data provide the clearest quantitative evidence to date of sensory-driven coevolution between the design and presentation of an iridescent visual signal. These findings support the idea that the angular visibility afforded by limited-view iridescence contributes to biased signal delivery. They also implicate temporal signal dynamics as a potentially important aspect of signal content, which could be profitably studied in species whose males exhibit limited-view sexual signals. *Hypolimnas* butterflies offer great potential for directly testing this hypothesis using wing transplantation techniques to manipulate signal directionality independently of brightness. Excellent opportunities also exist for quantifying the patterns of variation in finer scale features of both signal and display (such as peak UV reflectance angle, vertical courtship positioning, body roll, etc.), and for exploring the covariance between such factors among different males and/or their individual courtships.

## DATA ARCHIVING

The doi for our data is 10.5061/dryad.3hk2v.

## Supporting Information

Disclaimer: Supplementary materials have been peer-reviewed but not copyedited.

**Figure S1**. Schematic diagram of the reflectance spectrometry setup used for measuring the iridescent signal of male *Hypolimnas bolina*. **Figure S2**. Diagram of the male (below)–female (above) courtship positions “simulated” during reflectance spectrometry.
