# Supplementary material for: Signal design and courtship presentation coincide for highly biased delivery of an iridescent butterfly mating signal
Source: Evolution. 2014 Dec 3;69(1):14–25. doi: 10.1111/evo.12551 (PMC4312914; doi:10.1111/evo.12551)
Supplement: Supplementary file 1 [file evo0069-0014-sd1.pdf]

Online supporting information for

Signal design and courtship presentation coincide for highly biased delivery of an iridescent butterfly mating signal

Thomas E. White [A, D], *Jochen Zeil* [B], *Darrell. J. Kemp* [A, C]

[A] Department of Biological Sciences, Macquarie University, North Ryde 2109, NSW, Australia

[B] ARC Centre of Excellence in Vision Science, Research School of Biology, The Australian National University, Biology Place, Bld. 46, Canberra, ACT, 0200, Australia.

[D] Corresponding author.

Email: thomas.white026@gmail.com

This file includes:

Supplementary methods

Supplementary figures S1 & S2

Supplementary Methods

High-speed video recording

The greenhouse in which recording took place is covered by plastic roofing that transmits 60 % of incident light between 400-700 nm and ~ 20 % between 300-400 nm. The reduction in UV light is unlikely to affect courtships as male behaviour is highly ritualized (Rutowski 1992; Stride 1957), and the sexual signal reflects substantial amounts of light above 400 nm (figure 1, main text).

The complete analysis of high-speed video was a multi-stage process. First, we imported the raw .AVI files into the open-source video processing utility VirtualDub (copyright by Avery Lee, phaeron@virtualdub.org) where we separated out courtship and high-quality regular flight sequences, before exporting them as .jpg image sequences. We then used *digilite*, a program created in MATLAB (2011) by Jan Hemmi and Robert Parker of The Australian National University, to manually mark the x-y coordinates of points of interest frame-by-frame. We analysed the resulting coordinate data using custom written scripts in MATLAB after first converting the coordinate data to metric units (since coordinate data are initially stored as pixel values) using a scale based on male body length. Individual analyses progressed as follows.

Wingbeat frequency was calculated by plotting wingspan, defined as the length (in mm) between distal forewing tips, against time, and simply counting the number of wingbeats completed per time unit. Although a fast-Fourier analysis would be preferable for identifying the dominant frequency in such a signal, the method requires a minimum signal length for robust results in a noisy system, and several courtship sequences were too short (Brigham 1998).

Peak wingbeat amplitude, defined as the maximum angle created between the male wing and horizontal plane, was calculated using the formula

$$A = 90 - ((arcsin\left(\frac{(0.5 \times W)}{F}\right)) \times \left(\frac{180}{\pi}\right) \qquad (1)$$

where A is the peak amplitude in degrees, W is the mean male wingspan, and F is the mean male forewing size measured from distal tip to abdomen. For each of 28 courtship and 30 regular flight sequences, we calculated the amplitude of every up- and down-stroke of each wingbeat. Since equation (1) cannot distinguish between an up- and down-strokes within a wingbeat we visually identified the direction of the first stroke in the original footage, and assumed that the subsequent strokes progressed as an alternating cycle.

To calculate and plot the position of males relative to females during courtship in two-dimensions, we first subtracted the x-y coordinates of the male head from the x-y coordinates of the female head, which centred the male coordinate system on the female at 0, 0 in the x-y plane. We then used a rotation matrix to further transform the male x-y coordinates so that they are now expressed relative to the longitudinal body axis of the female at zero degrees. This was achieved according to the formula:

$$x' = x\cos\theta - y\sin\theta \qquad (2)$$

$$y' = x\sin\theta + y\cos\theta$$

, where x' and y' are the transformed coordinates of the male head, x and y are the raw male head coordinates, and θ is the orientation, in radians, of the female relative to the x axis.

Model testing

To normalise these data and allow for statistical testing, we generated 5642 random x-y coordinates (equivalent to the number of coordinates actually recorded), plotted them against each of the six contour maps, and extracted the corresponding signal intensity values (i.e., z values). We took the mean of these signal intensity values for each model, which is essentially a measure of the area under the surface in each of the six models. We then divided every actual signal intensity value by the mean of the randomised signal intensity values across each of the six models, and took the mean of the now-normalised signal intensity values. This process has the ultimate effect of normalising the recorded data within the context of each model.

References

Brigham E. (1988) *Fast Fourier Transform and Its Applications* (Prentice Hall, London).

MATLAB (2011) (The MathWorks Inc., Natick, Massachusetts), 7.12.0.

Rutowski R.L. (1992) Male mate-locating behavior in the common eggfly, *Hypolimnas bolina* (Nymphalidae). *J. Lep. Soc.,* 46, 24-38.

Stride G.O. (1957) Investigations into the courtship behaviour of the male of *Hypolimnas misippus L.* (Lepidoptera, nymphalidae), with special reference to the role of visual stimuli. *Brit. J. Anim. Behav.* 5, 153-167.

Supplementary Figures

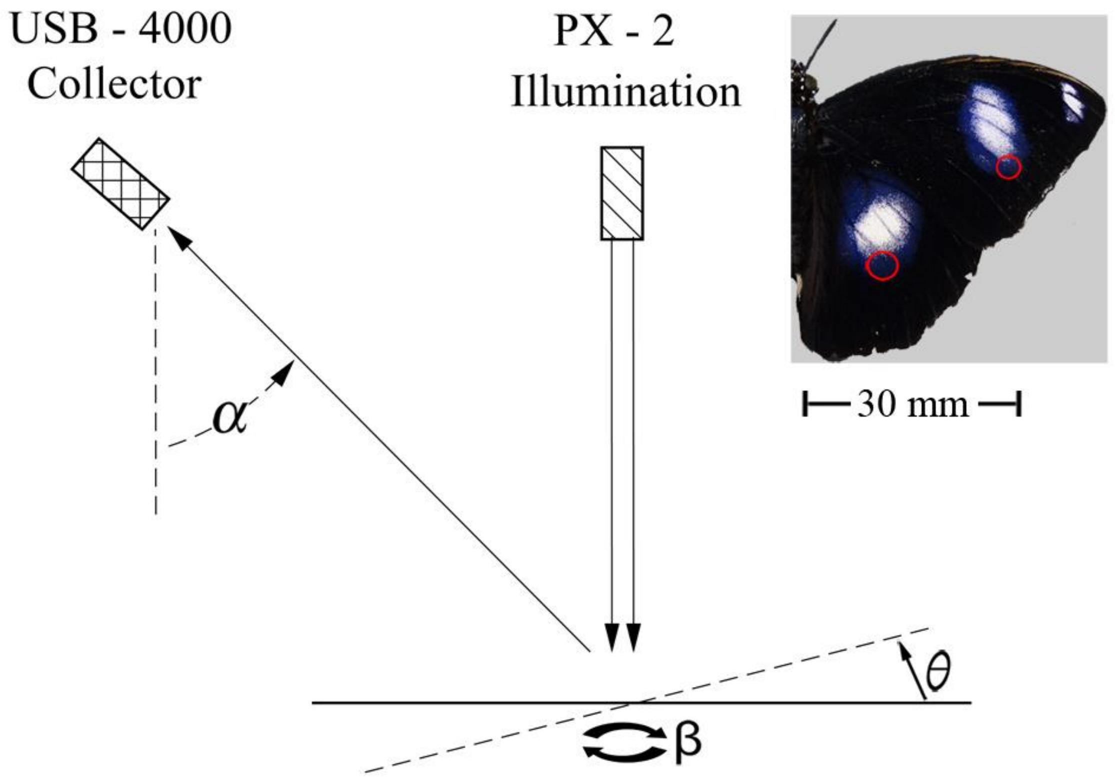

Figure S1. Schematic diagram of the reflectance spectrometry setup used for measuring the iridescent signal of male *Hypolimnas bolina.* Illumination was provided normal to the wing surface by a PX-2 pulsed xenon light source. An Ocean Optics USB-4000 photospectrometer collector was positioned to capture input at angle α from vertical, which is determined by the 3-d male-female position being 'simulated' (and corresponds to angle α in figure S2). Wings were set on a universal stage and rotated in a plane measured by the angle β to adjust body orientation, and were tilted on the long axis at angle θ to simulate five wing amplitudes (-40°, -20°, 0°, 20°, 40°). The image of a male common eggfly's wing (inset) indicates the fore- and hind-wing areas sampled for all four wings of twenty individuals.

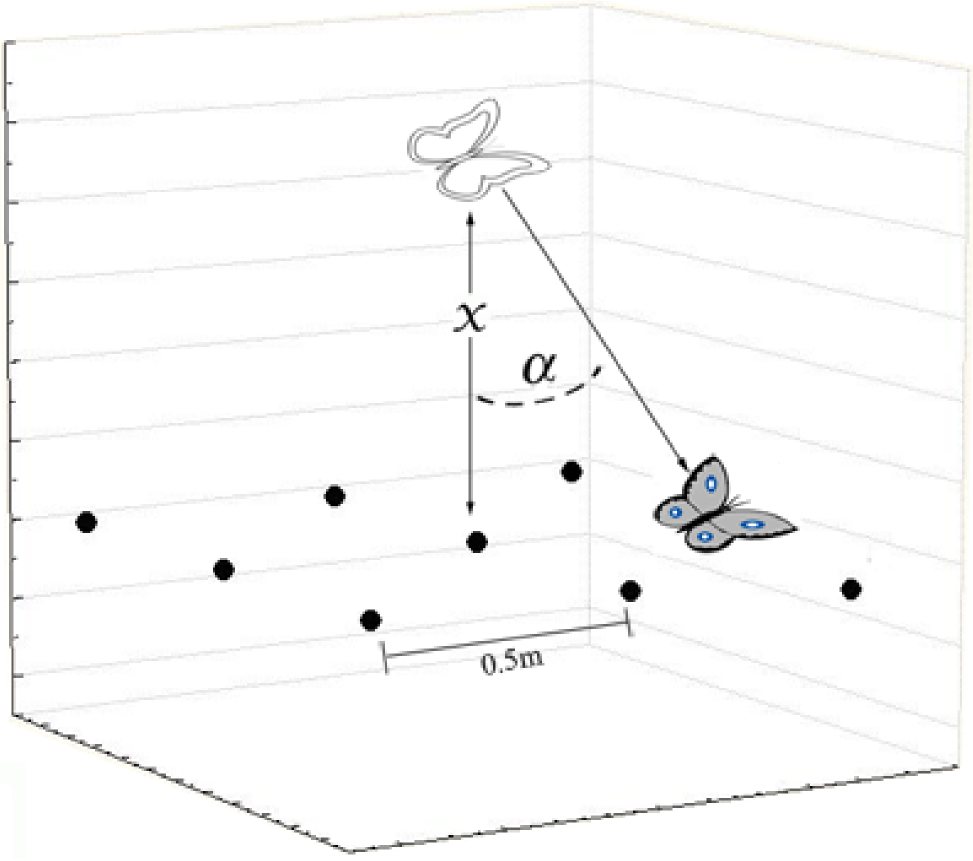

Figure S2. Diagram of the male (below) – female (above) courtship positions ‘simulated’ during reflectance spectrometry. Nine male positions at x = 0.2 m were considered. The angle α represents the angle between female receiver and male signaller, and corresponds with α in figure S1. In each position male body roll, pitch and yaw was held constant, illumination was assumed as normal to the wing surface, and wings were rotated through five angles (-40°, -20°, 0°, 20°, 40°). Distances between points are 0.5 m in the x-y plane, defining a maximum courtship area of 1 x 1 x 0.2 m.
